# A Low-Cost Real Color Picker Based on Arduino

**DOI:** 10.3390/s140711943

**Published:** 2014-07-07

**Authors:** Juan Enrique Agudo, Pedro J. Pardo, Héctor Sánchez, Ángel Luis Pérez, María Isabel Suero

**Affiliations:** 1 University Center of Merida, University of Extremadura, Sta. Teresa de Jornet, 38, Mérida 06800, Spain; E-Mails: jeagudo@unex.es (J.E.A.); sasah@unex.es (H.S.); 2 Physics Department, University of Extremadura, Avda. Elvas s/n, Badajoz 06006, Spain; E-Mails: aluis@unex.es (A.L.P.); suero@unex.es (M.I.S.)

**Keywords:** color, colorimetry, Arduino, Matlab

## Abstract

Color measurements have traditionally been linked to expensive and difficult to handle equipment. The set of mathematical transformations that are needed to transfer a color that we observe in any object that doesn't emit its own light (which is usually called a color-object) so that it can be displayed on a computer screen or printed on paper is not at all trivial. This usually requires a thorough knowledge of color spaces, colorimetric transformations and color management systems. The TCS3414CS color sensor (I2C Sensor Color Grove), a system for capturing, processing and color management that allows the colors of any non-self-luminous object using a low-cost hardware based on Arduino, is presented in this paper. Specific software has been developed in Matlab and a study of the linearity of chromatic channels and accuracy of color measurements for this device has been undertaken. All used scripts (Arduino and Matlab) are attached as supplementary material. The results show acceptable accuracy values that, although obviously do not reach the levels obtained with the other scientific instruments, for the price difference they present a good low cost option.

## 1. Introduction

The world around us is perceived through the senses, and of these, sight is the one which contributes more information to the human brain in its human-environment interface task. Sight gives us information through light, its intensity and color. Despite the complex processing that is carried out in human neurons from the moment the light reaches the rods and cones, this was the first human sense to be mathematical modeled through its own space of representation and measurement [1]. In 1931, the International Commission on Illumination defined the standard colorimetric observer [2] which sought to represent the average human being in terms of color vision in order to determine and identify a color based on a mathematical coordinates. Despite undergoing constant evolution since then, linked to the constant advances in knowledge of human visual physiology and psychometrics, the origin of complex colorimetric transformations performed to represent a color in an independent representation space are still based on this colorimetric standard observer.

These complex colorimetric transformations and concepts such as standard observer or CIE 1931 space contrasts with the ease with which anyone used to working with digital devices that specify a color by its RGB coordinates. However, this simplicity becomes difficult when these same people try to reproduce a particular color in a different electronic device such as a monitor or printer. This is because the spaces representing the digital color, RGB for monitors and digital cameras and CMYK for printers, are dependent color spaces of the device, that is, the same RGB color generates a simulation of a different color on a monitor depending on the primary colors that the monitor uses and the specific configuration of brightness, contrast, gamma and color temperature.

To solve this problem, critical in some professions such as graphic designers, photographers, *etc.* color management systems (CMS), as the name indicates, manage colorimetric transformations necessary to obtain an accurate color reproduction when using colorimetric profiles on each device [3]. These colorimetric profiles are separate files associated with each device that are able to reproduce or capture a color. They can also be integrated into the digital images generated by them. These files specify color from a point of view independent from the device, employing independent color spaces such as CIE 1931 or CIE Lab. These color management systems make it much easier but needs an external device that allows for real measurements of radiation emitted or reflected in each case, such as spectrophotometers or colorimeters.

Although there are many colorimeters on the market, that measure the color of any object in order to “digitize” it and begin these colorimetric transformations, they are usually quite costly in time and money. However, the development of modular low cost electronics platforms, such as Arduino [4], mbed [5] or raspberry PI [6] which can be equipped with all kinds of sensors, opens the door to the use of these devices for capturing colors.

This type of platform is spreading in research fields for low cost prototyping [7–11], allowing fast and easy development and extensive configuration options that are sometimes limited in commercial devices. Arduino [12] is a project whose aim is to provide simplicity in creating electronic projects. This platform is one of the most famous electronic prototype development boards because of its ease of use/programming and affordable price, leading to the so called democratizing technology [13] in which users create their own technology devices through the DIY philosophy.

The aim of this paper is to explore the possibilities of a prototype based on Arduino and the TCS3414 color sensor as a color capturer of solid objects—what we have called color picker—and the creation of software for management and control of color in a simple way. The results and the software developed are provided as supplementary material.

This article is organized as follows: In Section 2, a theoretical introduction of different color spaces is made, how they are generated and what their advantages and disadvantages are. In Section 3 the prototype and its characteristics are presented. In Section 3, the experiments and device possibilities are explained. In Section 4 we detail the calibration process and software development. Section 5 concludes with the work undertaken as well as the future outlook for this work.

## 2. Color Spaces

Kuehni [14] defines color spaces as three-dimensional geometric spaces with axes appropriately defined so that symbols for all possible color human perceptions fit into them psychologically ordered. In this space each color perception is represented as a point. From this definition, note each color that humans can perceive is defined by a single point in this three-dimensional space and corresponding the ordering of these points to the visual perception. This is important since uninitiated people in colorimetry associate color with the wavelength of light radiation that reach the eye but, for example, the same yellow color of the luminous radiation of a sodium lamp of 589 nm can be achieved by adding in suitable proportions of two lights, one green 540 nm and one red 620 nm. Under this point of view, the human visual system is trivariate and space of the electromagnetic radiation has infinite variance. Furthermore, if the geometric distance between two colors represented in a vector space corresponds to the color difference perceived by the human, the color space is said to be uniform.

### 2.1. Device-Independent Color Spaces

**Figure 1.**
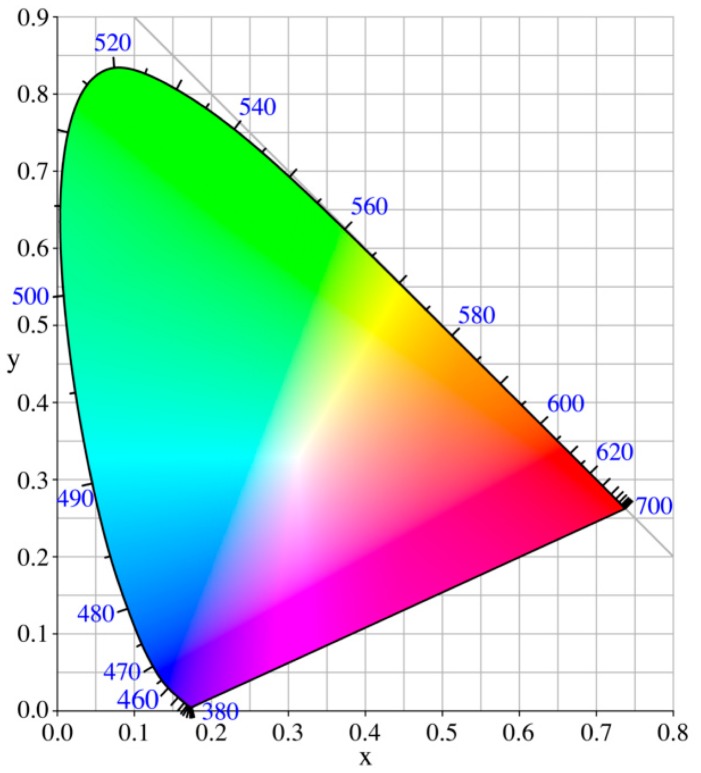
CIE 1931 chromaticity diagram.

The first color space used as reference system universally accepted was XYZ tristimulus space, standardized by CIE in 1931 [2]. This space arises directly from the sensitivity of the three types of cones that are found in the human retina and various visual properties specified by mathematical laws known as the Grassmann's laws [15] and define it as Euclidean space. Since it was very difficult to represent a three-dimensional space graphically, a two dimension diagram is known as chromaticity diagram CIE 1931 xy (Figure 1) where chromaticity coordinates x,y are obtained by normalization x = X/(X + Y + Z); y = Y/(X + Y + Z) allowing dispensed with the third coordinate z since z = 1 − x − y. Furthermore, as in the definition of tristimulus space as the chromaticity diagram, took advantage to introduce certain boundary conditions which eliminate the possibility to appear negative coordinates and for illuminant equi-energy E had as chromaticity coordinates (x,y) = (0.33,0.33).

Despite the progress that led to the existence of the CIE XYZ color space, and its associated chromaticity diagram (CIE1931 xy), this space does not have the second necessary property of a good color space: the correspondence of visual perception in terms of distances (color differences), so that in 1976 another color space is developed, emanating from the first, called CIE LAB [16] in which this aspect of the color differences as well as the independence of the target type used as illuminant are improved.

**Figure 2.**
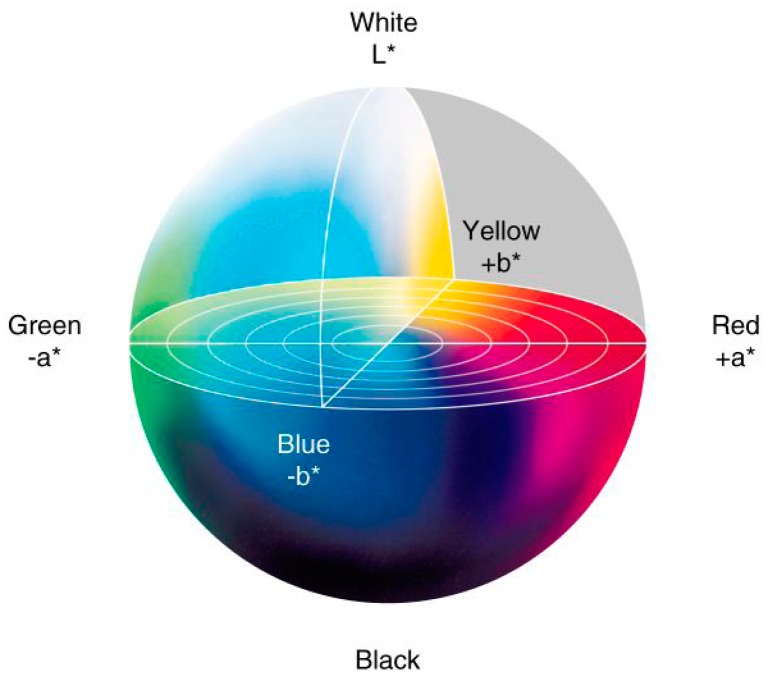
CIE LAB 1976 color space.

This space has an associated chromaticity diagram which corresponds to a color circle in a*b* plane centered at the origin (Figure 2).

### 2.2. Device-Dependent Color Spaces

Opposite to color spaces described above, there are color spaces associated with different electronic devices such as RGB, associated with screens and cameras, and CMYK spaces associated with printers and offset machines. These spaces are used to represent the colors that a device can display or capture depending on source sensitivity curves for each device or primary stimulus depending on the case. Therefore, it does not serve to communicate a color unequivocally by its own and must be connected with independent color space through correct chromatic device characterization, with which this paper deals.

## 3. Prototype

**Figure 3.**
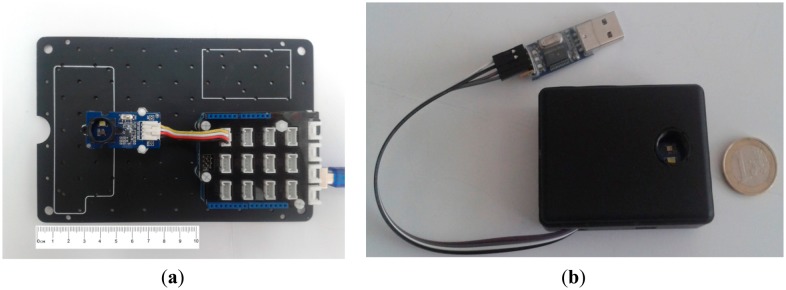
Color picker prototype prototype (**a**) and portable version (**b**).

In order to build the prototype, we have used an Arduino Uno with a shield Grove—Base Shield V1.3 and a color sensor Grove—I2C Color Sensor (Figure 3a), the overall cost of the system is less than 70$. Arduino Uno is the basic board within the existing Arduino family, based on the ATmega328 chip. It has 14 digital input/output pins (of which six can be used as PWM outputs) and six analog inputs. The ATmega328 included also supports I2C and SPI communication. It contains everything needed to start developing your own prototype; simply connect it to a computer with a USB cable and program it. Besides, a new prototype based on an Arduino Mini for a higher portability with equal characteristics has been developed (Figure 3b).

The Grove system [17] developed by Seedstudio [18] consists of an Arduino shield and modules with standardized connectors. The shield allows for easy connection of Arduino pinout from the Grove modules. Each one comes with demo code and documentation to help you use it easily. The base shield 1.3 has 8 connectors for digital input/output, 4 analog input connectors and four I2C connectors.

The selected module, Grove–I2C Color Sensor, uses the I2C serial protocol to communicate with Arduino. This module contains a Color Light-to-Digital Converter TCS3414cs sensor from the manufacturer ams AG [19]. According to the sensor manufacturer's specifications, the TCS3414 digital color light sensor is designed to accurately derive the color chromaticity and the luminance (intensity) of ambient light as well as provide a digital output with 16-bits of resolution. This device includes an 8 × 2 array of filtered photodiodes, analog-to-digital converters and control functions on a single integrated circuit. Of the 16 photodiodes, four have red filters, four have green filters, four have blue filters, and four have no filter (clear). The applications suggested by the manufacturer are: Light color temperature measurement, RGB LED Backlight control, RGB LED consistency control, Health fitness, industrial process control or use in medical diagnostic equipment.

**Figure 4.**
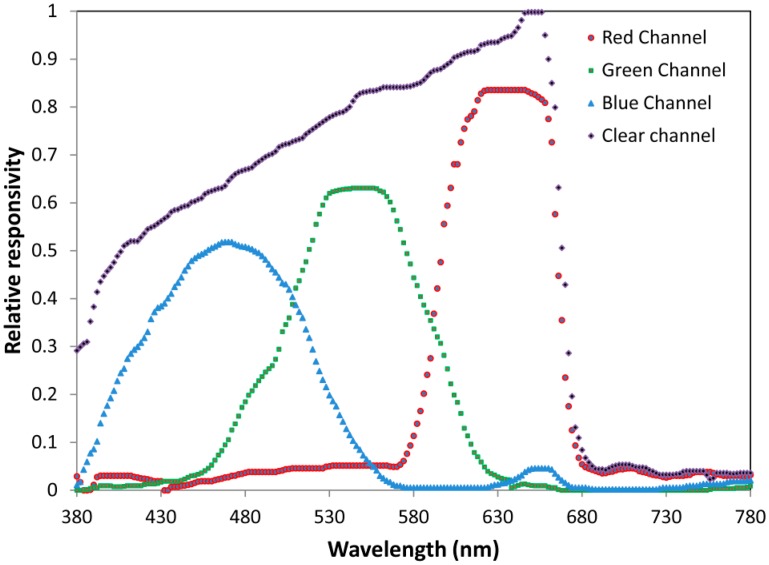
Spectral responsivity of the four channels.

Spectral response curves of the four channels of the sensor can be seen in Figure 4. The device has 16-Bit Digital Output with I2C at 400 kHz, programmable Analog Gain and Integration Time Supporting 1,000,000-to-1 Dynamic Range.

In order to control the device from Matlab we have created a simple command protocol via a serial port in the Arduino Board. The TCS3414 sensor has a timing register that controls the synchronization and integration time of the Analog-to-Digital-Conversor (ADC) channels. The Timing Register settings apply to all four ADC channels and it has three working modes. The default mode is a free-running mode in which one of the three internally-generated Nominal Integration Times (12, 100, 400 ms) is selected for each conversion. The second mode is a manual start/stop integration mode through a serial bus using ADC_Enable field in the control register. The third mode is based on the integration times preset in the free-running mode but synchronized with an external signal, allowing synchronization measures for pulsed sources.

The aim of the protocol therefore is controlling these operation modes and their different options. This serial communication protocol is based on alphanumeric characters to allow its remote and automatic execution to perform the checks that we will see in the following sections. The complete code is attached as supplementary material.

The protocol is based on a string with capitalized characters separate by blank spaces. The first character is always *R* to indicate that we will work remotely. Then you must specify the operating mode, choosing between *F* for free-running, *M* for manual or *A* for automatic. Next, for the three modes, the gain values (between 1 and 4) and the prescaling values (1 to 4) are fixed, always separating individual values by a space. In the case of free-running and manual modes, the integration time must be set: 12, 100, 400 ms, with a value between 1 and 3. In automatic mode, the system sensor + Arduino calculates the integration time required to obtain a constant level of response in the green digital channel. As an explanatory example, the “*R F 1 1 3*” sequence corresponds to the free-running mode with gain 1, prescaler 1 and integration time 400 ms. The “*R M 2 1 1*” sequence corresponds with manual mode with gain 2, prescaler 1 and integration time 12 ms.

## 4. Experimental Procedure

In order to measure the potential of our low cost electronic system, in terms of color measurement capabilities, we have undertaken a series of experiments and actions which are then explained.

### 4.1. Starting Setup

To test the capabilities of this system we have followed the manufacturer's instructions, the library and sample code provided by the manufacturer of the Grove module needed to run it using Arduino and conducted the first color measurements using the calibration parameters provided by the manufacturer's example. In these color measurements, we observed a number of inconsistencies between the measures obtained chromaticity and objects used as samples and when looking for the source of these inconsistencies we detected an error in the address memory of one of the two bytes of the red channel. Once this problem was fixed, we modified the sample code and the library and then started to obtain reasonable results that allowed us to continue with the experiments.

### 4.2. Linearity in the Channels Response

One common source of error in the measurements provided by an electronic device is the nonlinearity in the response of the digital channels that exist in the device. When this occurs, there are solutions based on artificial neural network models generation that improve the color signals from the sensor [20,21]. In this particular case, the TCS3414CS sensor has three channels, R, G, B and a fourth channel that is called Clear in which the sensor is exposed to radiation without any filter except an IR filtering component. To test the linearity of these channels an experimental device based on a video projector connected to a computer that generates light simulations of different RGB values projected onto the sensor. Also, together with the electronic device equipped with the sensor, a diffuse reflectance pattern [22] was produced on which the chromaticity coordinates of the light stimulation were measured using a Minolta CS-2000 tele-spectroradiometer controlled by the same computer.

**Figure 5.**
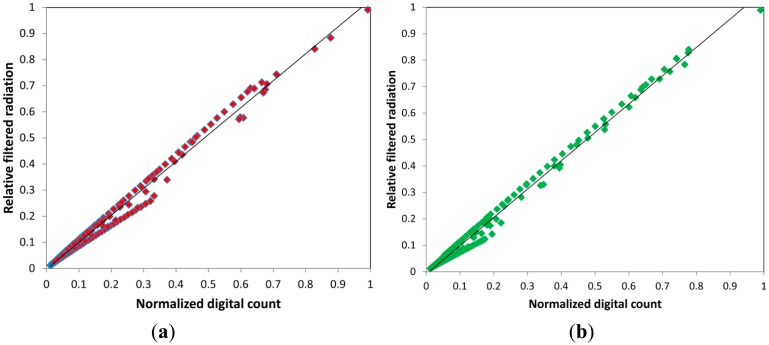

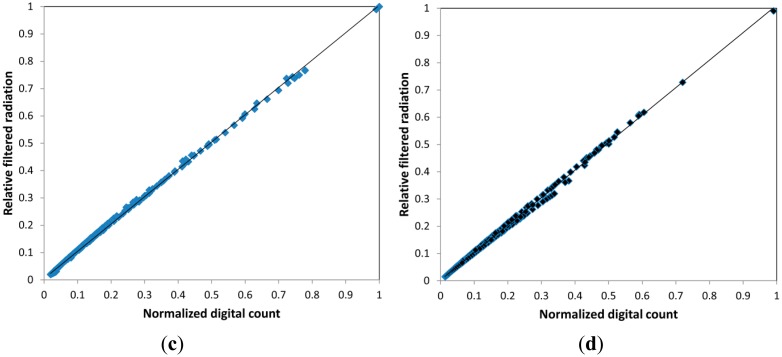
Normalized digital count VS. Relative filtered radiation received for the four channels (**a**) Red; (**b**) Green; (**c**) Blue; (**d**) Clear.

This measuring instrument has a spectral measurement range between 380 and 780 nm and accuracy in the measurement of CIE 1931 xy chromaticity of x = 0.0015 y = 0.001. Using proprietary software developed in Visual Basic and automatic control of a light stimulation generator, the test device measurement and reference measurement device, we conducted a study of the linearity of the RGB channels of the color sensor as graphically displayed in Figure 5. In all the channels, the adjustment in the linearity obtained exceeds Pearson's correlation coefficient of R > 0.99.

### 4.3. Influence of Integration Mode on the Fluctuation of the Measurement

As we mentioned, the TCS3414cs sensor has three working modes for controlling the color capture. In order to test how to use the sensor, several series of measurements were performed to check the operation of the first two modes, the third one is not applicable in our case. In the case of the free-running mode an integration time of 400 ms is set so the simulation used does not saturate the sensor with this integration time. In the second case, an algorithm is designed, that along with two previous measurements, calculates the integration time needed to maintain the sensor at 80% of its maximum response. With these two run modes the calibration procedure was performed, including a measure of the white reference target (in this case the white of a video projector), touring the RGB channels of the projector from 0 to 255 in steps of 5 units and performing measurements of 50 RGB values randomly generated.

Through this calibration process a 3 × 3 matrix is obtained that relates the RGB values provided by the sensor with XYZ tristimulus values needed to specify the color in a separate space, in this case the CIE 1931. As a method of obtaining this matrix the Microsoft Excel Solver tool was used, using the average chromaticity error of the measurements as an optimization parameter, excluding the random types which have been left to assess the accuracy of the measurements. Similarly, we used a second optimization parameter based on the absolute error of the XYZ tristimulus values. Table 1 shows the summary of the errors obtained on 50 random values measured in the two operating modes and with the two optimization methods, although in the case of the dynamic integration time, always looking for a same level of response does not make sense for the second type of adjustment.

In light of Table 1, the optimum integration method for this type of device is the free-running mode based on the internal clock of the sensor that ensures less error in chromaticity and tristimulus values. Therefore the final experiment has been conducted in this mode. The error in chromaticity obtained is usually greater than the error provided by commercially produced scientific measuring instruments but in line with what is expected of such low cost devices.

## 5. Results and Discussion

In the light of the preliminary results, the lowest average error in chromaticity measured is 0.005 units in the CIE 1931 chromaticity diagram. This means that dedicating this device to measuring correlated color temperature CCT, as indicated by the manufacturer's data sheet, would not be an optimal use for as in these types of measurements chromaticity accuracy is crucial. However, these results place this device in a position to be used as a good color meter, especially in relation to non-self-luminous solid objects, by taking advantage of the high power white LED that can be used as light source incorporated into the Grove model. These color measures could also do with any imaging device based on CCD or CMOS sensors if they provide access to raw images and have a good stability and linearity. It would also be necessary to include a light source that would use this device to measure colors by reflection, like Arduino based prototype presented here, and prepare a carrier fixed to keep the distance and angle between source, sample and sensor. These requirements are supported by medium-high range mobile phones of latest generation but its integration with the software for subsequent colorimetric transformations require some knowledge of more advanced programming and little changes in the mobile phone hardware. In our case, we have designed specific software based on matlab that automates the complex set of colorimetric transformations that are needed once the color is captured (picked) until it is displayed on the screen of a digital device. This code is also attached as supplementary material so that it can be used or modified by users.

### 5.1. Colorimetric Transformations

**Figure 6.**
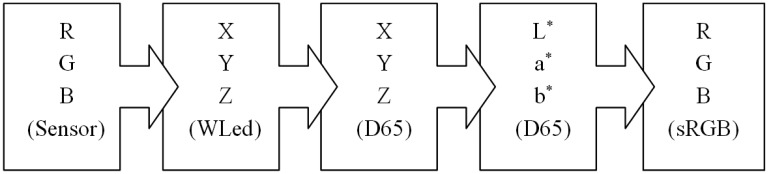
Colorimetric transformations.

The colorimetric transformations are necessary in order to display a color captured from a real object on the screen of a digital device with the most accurate appearance begin with the transformation of RGB color space captured by the sensor to XYZ space by calibrating the sensor. Afterwards we need to transform the tristimulus values obtained with the device's light source to appropriate values for a reference illuminant such as D65. This step attempts to alleviate capture errors as far as possible when using a different source to the reference illuminant, but inevitably introduces an error. Then they have to be converted from the XYZ values for the D65 illuminant to CIE Lab space values, as they are widely used due to their greater uniformity. Finally an RGB coordinates transformation to the output device needs to be undertaken and then the color can be displayed. In this last step the characteristics of the color management systems that are usually responsible for managing color in electronic devices and used as a reference for RGB space is taken into account [23]. Figure 6 summarizes graphically the transformations needed.

### 5.2. Calibration

**Figure 7.**
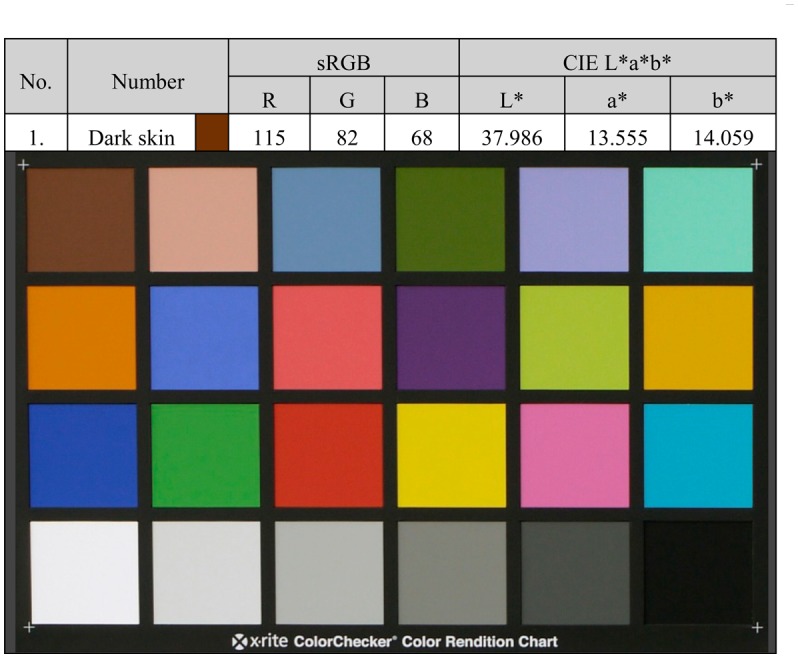
Chromatic chart ColorChecker and chromatic values in CIE Lab space and sRGB.

To simplify the calibration process used in preliminary experiments and adapted to the new objective of measuring solid colors, we have designed a calibration process based on the most widely recognized color card, the X-Rite Color Checker [24].This color card consists of 18 chromatic samples and 6 achromatic, and their chromaticities are provided by the manufacturer under the illuminant reference D65. Figure 7 shows a picture of the color chart with the values provided by the manufacturer for the first sample.

Anyone who possesses this color card only needs to measure the RGB response of each sample using the Arduino serial communication and enter the coordinates of the RGB color rows in a text file. To facilitate the process of obtaining the calibration matrix, we have implemented a matlab script called *Calibration* along with necessary supporting files that generate this matrix and store it in native matlab format. All of this code is attached as supplementary material.

### 5.3. Color Management Software

**Figure 8.**
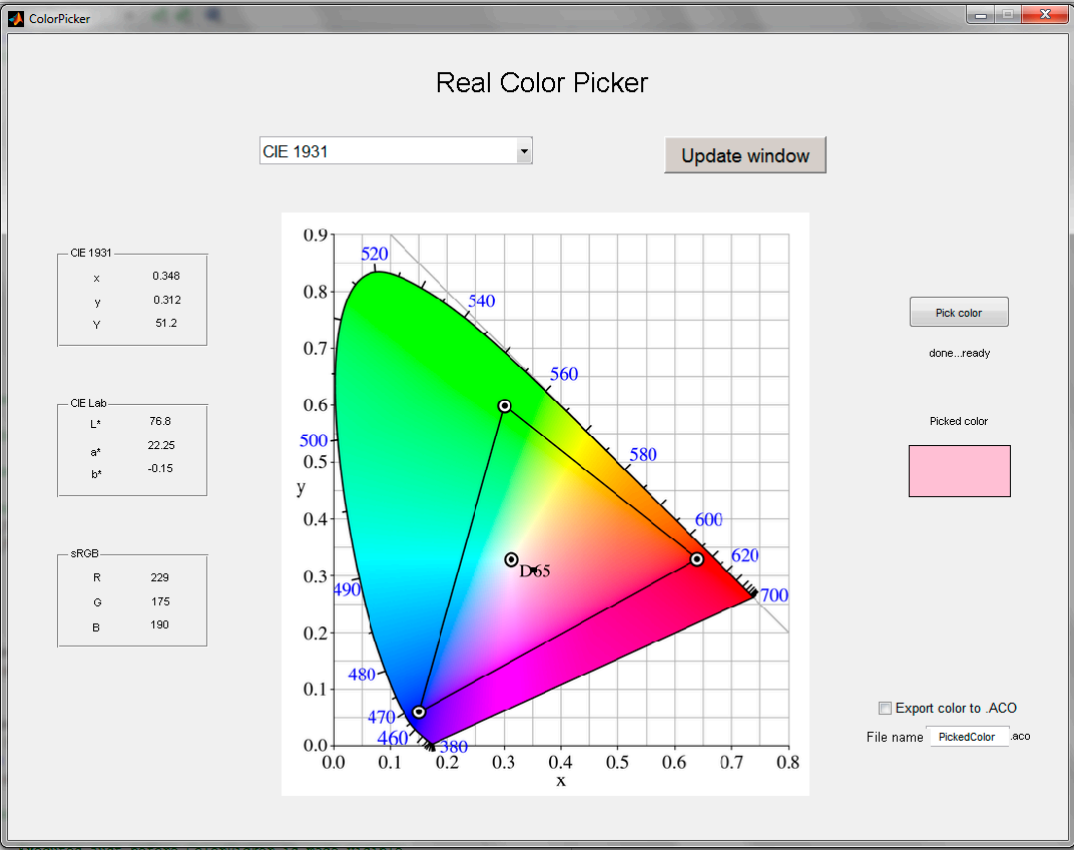
GUI of the software implemented in Matlab.

In order to automate the complex process of color capture and subsequent colorimetric transformations we have implemented a matlab script along with a graphical interface. Once the calibration process is completed by placing the color sensor on the object and clicking on the “pick color” button, the program takes care of setting the native RGB coordinates of the measuring instrument, transforming them to an independent color space, either CIE 1931 or CIE Lab, presenting the chosen chromaticity diagram and calculating the RGB color coordinates within the sRGB standard color space. The sRGB color space is the most widely used and is delimited by the chromaticity of the primaries used by a triangle in the CIE 1931 diagram. The chromaticity of the white reference is also recorded, in this case the illuminant D65. In Figure 8, shows the triangle with all reproducible chromaticities in a display that complies with this standard.

We have also added two more options, selectable by a drop-down control, in which an image is displayed with an altered color profile embedded so that if a software application does not perform, the color management appears in green tones whereas if management is correct, the color appears in reddish tones, typical of a sunset. This image is also provided as supplementary material as it is considered very useful for detecting their use by CMS graphics applications.

To complement the software, a matlab script can export the captured color to a file in Adobe COlor file (ACO), which allows the graphic palette of the software to be imported into Adobe Photoshop and, if it is out of the sRGB colour triangle, transforms it to the closest reproducible color.

## 6. Conclusions and Outlook

The possibilities that modular electronics offers are endless and, in a large part, this diversity is based on the existence of very different types of sensors, actuators, *etc.*, their ease of use and low cost. In this case, the combination of Arduino, the Grove system and a color sensor accompanied by a high power white LED, can capture the color of any readily homogeneous solid object and display it on the screen of an electronic device in the most reliable way possible. The results indicate that this system of color measurement has similar accuracy to other electronic devices, however without the accuracy of purely scientific instruments, but enough for a non-professional user. This shows that today valid research prototypes can perform for very low cost, although without the accuracy of commercial ones, providing an alternative for limited budgets.

In addition, as all the components are open hardware and the software is open source anyone can use and/or adapt the work presented in this paper following the philosophy of open knowledge. Moreover, this accuracy could be improved if a white light source LED that simulates the spectrum of the illuminant D65 is used as reference white in most colorimetric transformations.

## Figures and Tables

**Table 1.** Average chromaticity and tristimulus error obtained by two optimization methods.

**Optimization Parameter**	**Lower Average Chromaticity Error**		**Lower Average Tristimulus Error**	

**Integration Time**	**Chromaticity Error**	**Relative Tristimulus Error**	**Chromaticity Error**	**Relative Tristimulus Error**
Internal fixed	0.005	0.03	0.03	0.04
Manually fixed	0.07	N.A.	0.07	N.A
